# Diagnostic Accuracy of Nipple Discharge Fluid Cytology: A Meta-Analysis and Systematic Review of the Literature

**DOI:** 10.1245/s10434-021-11070-2

**Published:** 2021-11-27

**Authors:** Natasha Jiwa, Swathica Kumar, Rishikesh Gandhewar, Hemali Chauhan, Vikneswaran Nagarajan, Corrina Wright, Dimitri Hadjiminas, Zoltan Takats, Hutan Ashrafian, Daniel Richard Leff

**Affiliations:** 1 Department of Surgery and Cancer, Imperial College London, St Mary’s Hospital, London, UK; 2 Royal College of Surgeons Ireland, Dublin, Ireland; 3 Northwest London Pathology, Imperial College Healthcare Trust, London, UK; 4 Department of Breast Surgery, Imperial College Healthcare Trust, London, UK

## Abstract

**Background:**

Nipple discharge is the third most frequent complaint of women attending rapid diagnostic breast clinics. Nipple smear cytology remains the single most used diagnostic method for investigating fluid content. This study aimed to conduct a systematic review and meta-analysis of the diagnostic accuracy of nipple discharge fluid assessment.

**Methods:**

The study incorporated searches for studies interrogating the diagnostic data of nipple discharge fluid cytology compared with the histopathology gold standard. Data from studies published from 1956 to 2019 were analyzed. The analysis included 8648 cytology samples of women with a presenting complaint of nipple discharge. Both hierarchical and bivariate models for diagnostic meta-analysis were used to attain overall pooled sensitivity and specificity.

**Results:**

Of 837 studies retrieved, 45 fulfilled the criteria for inclusion. The diagnostic accuracy of the meta-analysis examining nipple discharge fluid had a sensitivity of 75 % (95 % confidence interval [CI], 0.74–0.77) and a specificity of 87 % (95 % CI, 0.86–0.87) for benign breast disease. For breast cancer, it had a sensitivity of 62 % (95 % CI, 0.53–0.71) and a specificity 71 % (95 % CI, 0.57–0.81). Furthermore, patients presenting with blood-stained discharge yielded an overall malignancy rate of 58 % (95 % CI, 0.54–0.60) with a positive predictive value (PPV) of 27 % (95 % CI, 0.17–0.36).

**Conclusions:**

Pooled data from studies encompassing nipple discharge fluid assessment suggest that nipple smear cytology is of limited diagnostic accuracy. The authors recommend that a tailored approach to diagnosis be required given the variable sensitivities of currently available tests.

**Supplementary Information:**

The online version contains supplementary material available at 10.1245/s10434-021-11070-2.

Nipple discharge may arise from both pathologic and physiologic processes and accounts for 3 % to 9 % of referrals to the breast clinic, the equivalent of 16,000 to 48,000 presentations each year in the United Kingdom.^1^ Spontaneous single-duct discharge is widely accepted as a clinical sign warranting further investigation, often requiring surgical management in the form of a microdochectomy or total duct excision to acquire a definitive diagnosis.^2^ A rising incidence of breast cancer^3^ has led to an urgent need for the development of rapid, reliable, and cost-effective methods of diagnosing breast cancer. Importantly, in the midst of a SARS-Cov-2 global pandemic, with everchanging hospital policies limiting exposure to various parts of the hospital, restrictions on the number of diagnostic methods offered to patients and the need to avoid unnecessary surgical intervention, a single noninvasive point-of-care diagnostic test to exclude breast carcinoma has become increasingly important.

In current practice, patients presenting with nipple discharge undergo triple assessment (clinical assessment, imaging, and pathology), which can include cytopathology prepared as a nipple smear. Clinical investigation of patients with pathologic nipple discharge (PND), defined as spontaneous single-duct and often blood-stained discharge, includes mammography, ultrasonography, magnetic resonance imaging (MRI), and even galactography to direct visualization of the ductal system ± ductal lavage. Although a recently published meta-analysis^4^ compared the diagnostic accuracy of different imaging methods used for the investigation of PND, the capacity of cytology to interrogate PND comprehensively for both benign and malignant diagnoses is yet to be reviewed systematically.

Nipple-smear cytology, still currently performed in many breast centers around world, is used as part of the workup for patients presenting with PND. Its role as an early detection tool for asymptomatic women also has been investigated^5,6^ given the feasibility of nipple aspirate fluid production by massage,^7^ negative suction devices (automated or manual),^8^ or ductal lavage.^9^ The diagnostic utility of nipple fluid cytology has been deliberated over the years.^10–12^ To date, however, the diagnostic accuracy of nipple fluid cytology for both benign and malignant diagnoses has not been comprehensively quantified using meta-analytical techniques.

To this end, the primary aim of this study was to perform a systematic review and meta-analysis to compute the diagnostic accuracy of nipple discharge fluid cytology for symptomatic women presenting to the breast clinic. The secondary aim was to investigate the variations in the management of PND in terms of presentation, imaging, pathology, and surgery as well as the diagnostic accuracy of other methods including ultrasound, MRI, and ductoscopy.

## Methods

An electronic search using MEDLINE, EMBASE, and SCOPUS was performed until March 2020. Multiple methods were used to retrieve papers, namely, submitting requests through the authors’ academic institution and the British Library, writing to the editor of the journal, contacting the corresponding author, and placing requests through ResearchGate.

Search terms included “nipple discharge fluid” and “cytology” in all their forms. The following Medical Subject headings (MeSH) and key words were used in combination with AND/ OR operators: “nipple discharge” OR “breast” adjacent to “discharge” by up to three words OR “nipple” adjacent to “discharge” by up to three words AND cytodiagnosis OR cytoproliferation OR cytolog* OR cytodiagnos* OR papanicolaou. Title and abstract review then was performed according to the predefined inclusion and exclusion criteria defined in the following sections.

### Inclusion Criteria

Only clinical studies with primary data on the diagnostic accuracy of nipple discharge fluid cytology versus ductal histology were included. Foreign language studies were included if an English language translation was retrievable. Studies were included if they yielded diagnostic information on benign and/or malignant diagnoses from cytology and on pathologic nipple discharge of all clinical descriptions (i.e., single duct, blood-stained, clear). Regarding acquisition of fluid, studies that included direct expression of discharge as well as dutoscopy to retrieve a fluid sample were included if patients presented with pathologic nipple discharge.

### Exclusion Criteria

Studies were excluded if a full English text was not available, or if a translation of the text into English was irretrievable. All animal studies, case reports, and male breast cancer studies were excluded. Studies with pregnancy-associated breast cancer also were excluded, as well as papers reporting on brush cytology only.

### Study Quality

Study quality (Table 1, Supplement 1) was evaluated by two independent investigators (N.J and S.K) using the Quality Assessment of Diagnostic Accuracy Studies 2 (QUADAS-2) scoring system checklist.^13^ All QUADAS-2 questions were included in quality scoring, providing a maximum score of 14.^13^ Each question was given a score of 0, 1, or 2 depending on whether the question was unanswered, unclearly answered, or fully answered. For studies to be considered accurately conducted and analyzed, the they had to report patient demographics, the presenting complaint, a clear explanation of the methods of processing and analyzing the nipple fluid smear, and whether an operative histologic sample or core biopsy was used for comparison. Whether the cytopathologist was blinded to the clinical results also was documented.

**Table 1:** Malignant cytology: demographics and outcome data for malignant diagnoses

Author and year	No. of patients	No. of samples	Malignant sensitivity (relative Cn3/4/5)	Malignant PPV	Malignant NPV	Malignant sensitivity (absolute Cn5)
Alcock & Layer^37^	49	49	0.33	1.00	0	
Bauer et al.^38^	12	23	1.00			
Cabioglu et al.^39^	188	23	0.30	1.00	0	
Cabioglu et al.^20^	146	69	0.81	0.28	0.80	
Carty et al.^40^	56	56	0.75	0.50	0.50	
Çetin & Sikar^41^	111	95	0.77	0.19	0.83	
Ciatto et al.^42^	50181	3687	0.61	0.64	0.88	
Cilotti et al.^43^	67	67	0.83	0.23	1.00	0.60
Denewer et al.^44^	54	54	0.60	0.43	0.88	0.40
Dinkel et al.^16^	384	384	0.38	0.16	0.96	0.19
El-Daly and Gudi^18^	98	98	0.50	0.50	0.90	
Florio et al.^45^	1251	194	1.00	0.27	0	
Fung et al.^46^	840	176	0.83	0.43	0.78	
Funovics et al.^47^	134	134	0.69	0.25	0.92	0.08
Groves et al.^48^	338	329	0.47	0.88	0.96	0.47
Grunwald et al.^49^	15	15	0.33	0.25		
Hahn et al.^50^	33	32	0.05	0.50	0.29	
Hou et al.^51^	146	156	0.71	0.15	0.85	0.29
Hou et al.^52^	487	176	0.72	0.18	0.83	0.32
Hünerbein et al.^53^	101	45	1.00	0.60	1.00	0.67
Jacobs et al.^54^	11	8	0.50	0.50	0.67	
Kalu et al.^55^	160	89	0.78	0.12	0.93	
Kan et al.^17^	102	37	0.78	0.41	0.80	
Kaplan et al.^19^	50	50	0.50	0.25	0.80	0.10
Kjellgren^56^	39	39	0.67	0.17	0.94	
Kooistra et al.^12^	618	618	0.50	0.15	0.91	
Kuroi et al.^57^	19	19	1.00	0	1.00	
Lee^58^	165	174	1.00	0.67	1.00	0.53
Matsunga et al.^59^	323	80	0.35	1.00	0	
Montroni et al.^60^	915	634	0.76	0.33	0.80	0.19
Morrogh et al.^10^	416	37	0.69	0.55	0.71	
Ohlinger et al.^61^	214	134	0.23			
Pritt et al.^62^	395	44	1.00	0.33	1.00	0.67
Rimsten et al.^34^	80	80		0.13		
Simmons et al.^63^	108	34	0.38	0.30	0.79	0.13
Shen et al.^64^	415	166	1.00	0.64	1.00	
Walker and Sanclison^33^	135	25	1.00	0.38	1.00	
Yang et al.^65^	419	277	0.12			
Yamamoto et al.^66^	65	39	0.50	0.50	0.94	

Details of the studies in the meta-analysis required to calculate the pooled diagnostic values: patient numbers in each study, data parameters including the relative and absolute sensitivity, and positive and negative predictive values

*PPV* positive predictive value, *NPV* negative predictive value

### Data Collection

An independent assessment by two investigators (N.J and S.K) was conducted using Covidence systematic review software (Veritas Health Innovation, Melbourne, Australia).^14^ Any conflicts were discussed and resolved with an explanation of “yes,” “no,” or “uncertain.” All “uncertain” cases underwent full-text screening, and justification for inclusion or exclusion was documented within the system (Fig. S1) and discussed with senior authors (H.A and D.R.L).

Demographic and accuracy data from the included studies were recorded using a predefined spreadsheet (Excel). In particular, data were extracted on the first author and year of publication, number of patients, number of cytology samples, mean age, QUADAS-2 score, method of collection, sensitivity, specificity, true-positives, false-positives, true-negatives, false-negatives, and positive predictive values.

After data extraction, the studies were subdivided by their method of collection (e.g., ductal lavage, manual compression) for subgroup analysis of sensitivity and specificity by method. Benign cytology was classified as “benign” or “Cn2,” representing “cytology for nipple fluid” adapted from the five-number grading system for fine-needle aspirate cytology of breast tissue as follows: insufficient (C1), benign (C2), atypical/equivocal (C3), suspicious (C4), or malignant (C5). Atypical and malignant cytology (including ductal carcinoma *in situ* [DCIS]/lobular carcinoma *in situ* [LCIS]) was defined using the numeric grading system Cn3–5 to calculate a relative sensitivity and specificity with an accompanying diagnostic accuracy curve and using Cn5 only to calculate the absolute sensitivity. Further analysis was performed using Cn2–3 to denote a benign diagnosis and Cn4–5 to denote a malignant diagnosis (Table 1 in Supplement 2).

### Meta-Analysis

Sensitivity, specificity, true-positive, true-negative, false-positive, false-negative, and positive predictive value (PPV) of cytology results were assessed for each paper, creating an overall sensitivity and specificity for both benign and malignant diagnoses. Pooled diagnostic sensitivity and specificity were calculated using 33 of the 45 studies reporting benign outcomes and 39 of the 45 studies reporting malignant outcomes alike (all studies with sensitivities of 0 were excluded from the calculation). In addition, these papers were interrogated for all comparative imaging and diagnostic methods. In particular, the overall malignancy rate for blood-stained discharge as well as the pooled sensitivity and specificity of mammography, ultrasonography, MRI, and galactography (or ductography) all were calculated independently.

Summary estimates of sensitivity, specificity, and area under the curve (AUC) data were attempted using a bivariate model for diagnostic meta-analysis. Independent diagnostic metrics and their differences were calculated and pooled through DerSimonian and Laird random-effects modeling.^15^ This considered both between-study and within-study variances, which contributed to study-weighting. Study-specific estimates as well as 95 % confidence intervals (CIs) were computed and represented on forest plots. Statistical heterogeneity was determined by the *I*^2^ statistic whereby less than 30 % was low, 30 % to 60 % was moderate, and more than 60 % was considered high. Analyses were performed using Stata version 15 (Stata Corp LP, College Station, TX, USA). Probability values (*p* values) of 0.05 or lower were considered statistically significant.

## Results

**Fig. 1:**
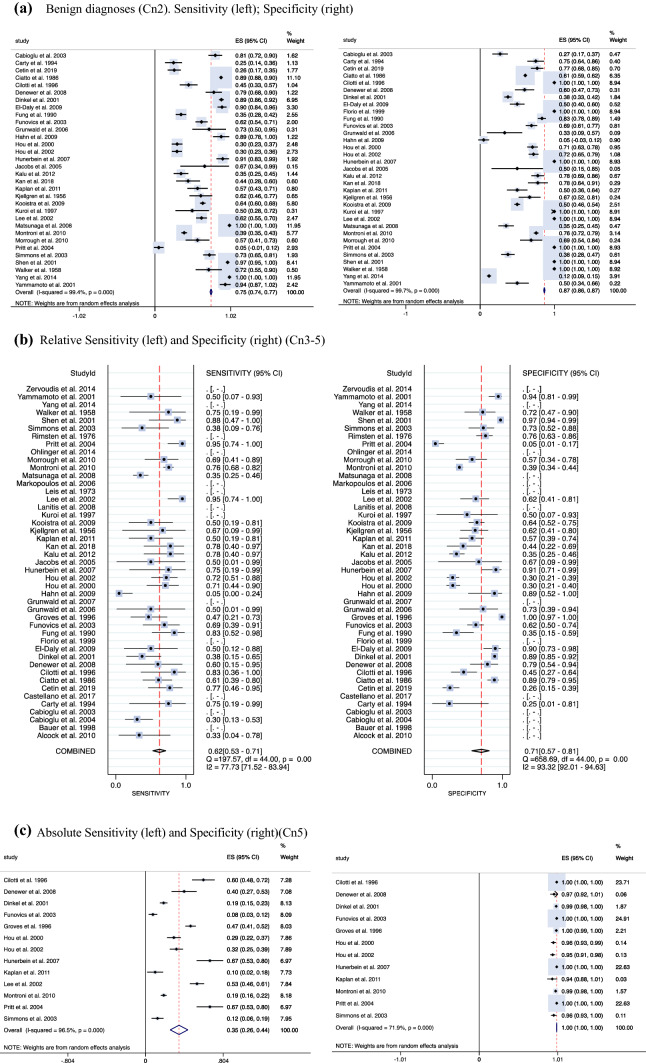
**A** Forest plots depicting the overall sensitivity (*left*) and specificity (*right*) of nipple discharge fluid cytology for patients with benign diagnoses classified as Cn2. **B1** Forest plots demonstrating the overall relative sensitivity (*left*) and specificity (*right*) of nipple discharge fluid cytology for patients with a malignancy (Cn3–5). **B2** Absolute sensitivity (*left*) and specificity (*right*) of nipple discharge cytology for Cn5 alone

For initial review, 837 studies were retrieved from the databases (PRISMA diagram; Supplement 1; Fig. 1). After the abstract and title review, 213 studies met the inclusion criteria for full text-review, with 168 studies excluded. The main reasons for exclusion were no English translation of the article (*n* = 70), lack of nipple discharge cytology data (*n* = 45), abstract only (*n* = 15), nipple discharge cytology data without gold standard comparison (*n* = 14), duplication of the dataset (*n* = 12), and merging of fine-needle aspirate cytology and nipple smear cytology (*n* = 7). Other exclusions ruled out patients not presenting exclusively with nipple discharge (*n* = 2), ductal lavage cytology with no simple nipple discharge cytology (*n* = 2), paper not available (*n* = 2), nipple aspirate fluid cytology rather than nipple discharge cytology (*n* = 2), case report (*n* = 1), and heterogeneous analysis of both male and female cytology data (*n* = 1).

The meta-analysis included 45 studies, all of which contained clinical data on the diagnosis acquired from nipple discharge cytology, which was correlated with their histology. The publication dates included in these studies ranged from 1956 to 2019. The mean or median age was available for 30 of the 45 studies, with an age range of 14 to 94 years. The mean age of the included patients was 48.74 ± 4.66 years.

**Fig. 2:**
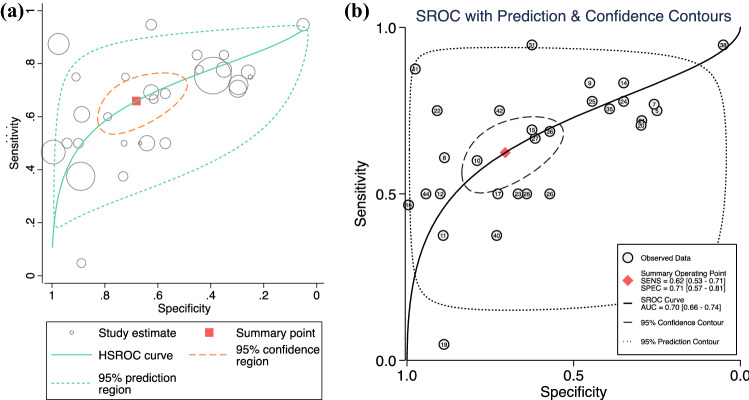
**A,B** Diagnostic accuracy curves illustrating both prediction and confidence contours, which demonstrate the relative sensitivity and specificity of nipple discharge fluid cytology with malignant diagnoses

Overall, the analysis included 8648 cytology samples. From the available data, sensitivity and specificity for nipple fluid smear cytology was either extracted or calculated. The computed relative and absolute sensitivity, PPV, and negative predictive value (NPV) for each study are included in Table 1 for malignant diagnoses. Table 2 presents the data for all non-cytologic diagnostic methods including sensitivity, specificity, PPVs, and NPVs. The diagnostic accuracy meta-analysis of nipple aspirate fluid showed a sensitivity of 0.75 (95 % CI, 0.74–0.77) and a specificity of 0.87 (95 % CI, 0.86–0.87) for a benign diagnosis (Cn2) (Fig. 1A). For breast carcinoma (Cn3/4/5), the meta-analysis showed a *relative* sensitivity of 0.62 (95 % CI, 0.53–0.71) and a specificity 0.71 (95 % CI,0.57–0.81) (Fig. 1B1). When only Cn5 cytology was considered, the absolute sensitivity of cytology was 0.35 (95 % CI, 0.26–0.44), and the specificity was 1.00 (95 % CI, 1.00–1.00) (Fig. 1B2). The overall diagnostic accuracy of nipple discharge cytology for a malignant diagnosis, including both prediction and confidence contours, is depicted in Fig. 2A and B, with the size of each circle representing the weight assigned to each study.

**Table 2:** Non-cytologic diagnostic methods: outcome data for imaging methods

Author and year	No. of patients	No. of samples	Sensitivity	Specificity	PPV	NPV
Mammography						
Alcock and Layer^37^	49	49		0		
Bauer et al.^38^		12		0.5		
Cabioglu et al.^39^	188	23				
Cabioglu et al.^20^	146	69	0.69	0.75	0.42	0.9
Çetin and Sikar^41^	111	95	0.17	0.96		
Fung et al.^46^	840	176	0.13	0.99		
Grunwald et al.^67^	65	58	0.38	0.92		
Kalu et al.^55^	160	89	0.33	0.82	0.78	0.38
Morrogh et al.^10^	416	37	0.18	0.94		
Ohlinger et al.^61^	214	134	0.57	0.33	0.58	0.32
Simmons et al.^63^	108	34	0.57	0.62	0.18	0.91
Ultrasound						
Cetin et al.^41^	111	95	0.66			
Grunwald et al.^49^	15	15	0.75		1	0.2
Grunwald et al.^67^	64	58	0.67			
Matsunaga et al.^59^	323	80	0.73			
Ohlinger et al.^61^	214	134	0.83		0.58	0.43
MRI						
Çetin et al.^41^	111	95	0.62	0.73		
Grunwald et al.^67^	64	58	0.65	0.25		
Kalu et al.^55^	160	89	0.65	0.73	0.73	0.57
Morrogh et al.^10^	416	37	0.7	0.44		
Ohlinger et al.^67^	214	134	0.83	0.12	0.61	0.36
Galactography						
Cabioglu et al.^20^	146	69	1	0.056	0.16	1
Grunwald et al.^49^	15	15	0.6	0	0.6	1
Grunwald et al.^67^	64	58	0.56	1		
Kalu et al.^55^	160	89	0.65	0.67	0.93	0.22
Kuroi et al.^57^	19	19		0.57		
Montroni et al.^60^	915	634	0.54			
Morrogh et al.^10^	416	37	0.79			
Ohlinger et al.^61^	214	134	0.8	0.44	0.58	0.7
Simmons et al.^63^	108	34	0	0.9	0	0.82
Yamamoto et al.^66^	65	39		0.82		
Alcock and Layer^37^	49	49			0.2	0.96
Cabioglu et al.^20^	146	69			0.18	0.9
Castellano et al.^68^	139	139			0.27	0.85
Çetin and Sikar^41^	111	95			0.21	0.92
Cilotti et al.^43^	67	97			0.19	1
Hou et al.^51^	146	156			0.25	0.91
Hou et al.^52^	487	176			0.83	0.43
Jacobs et al.^54^	11	8			0.25	
Kan et al.^17^	102	37			0.44	0.9
Kjellgren^56^	39	39			0.07	0.92
Lee^58^	165	174			0.21	
Leis^35^	259	259			0.15	0.93
Markopoulos et al.^36^	110	110			53.8	0.41
Matsunaga et al.^59^	323	80			0.25	
Montroni et al.^60^	915	634			0.26	0.82
Rimsten et al.^34^	80	80			0.08	0.98
Simmons et al.^63^	108	34			20	
Walker and Sanclison^33^	135	25			0.2	0.85

Includes studies carrying diagnostic data from imaging methods such as mammogram, ultrasound, MRI, galactography, blood, and malignancy. Data parameters include (where available or raw data was present to calculate) sensitivity, specificity, PPV, and NPV.

*PPV* positive predictive value, *NPV* negative predictive value, *MRI* magnetic resonance imaging

**Fig. 3:**
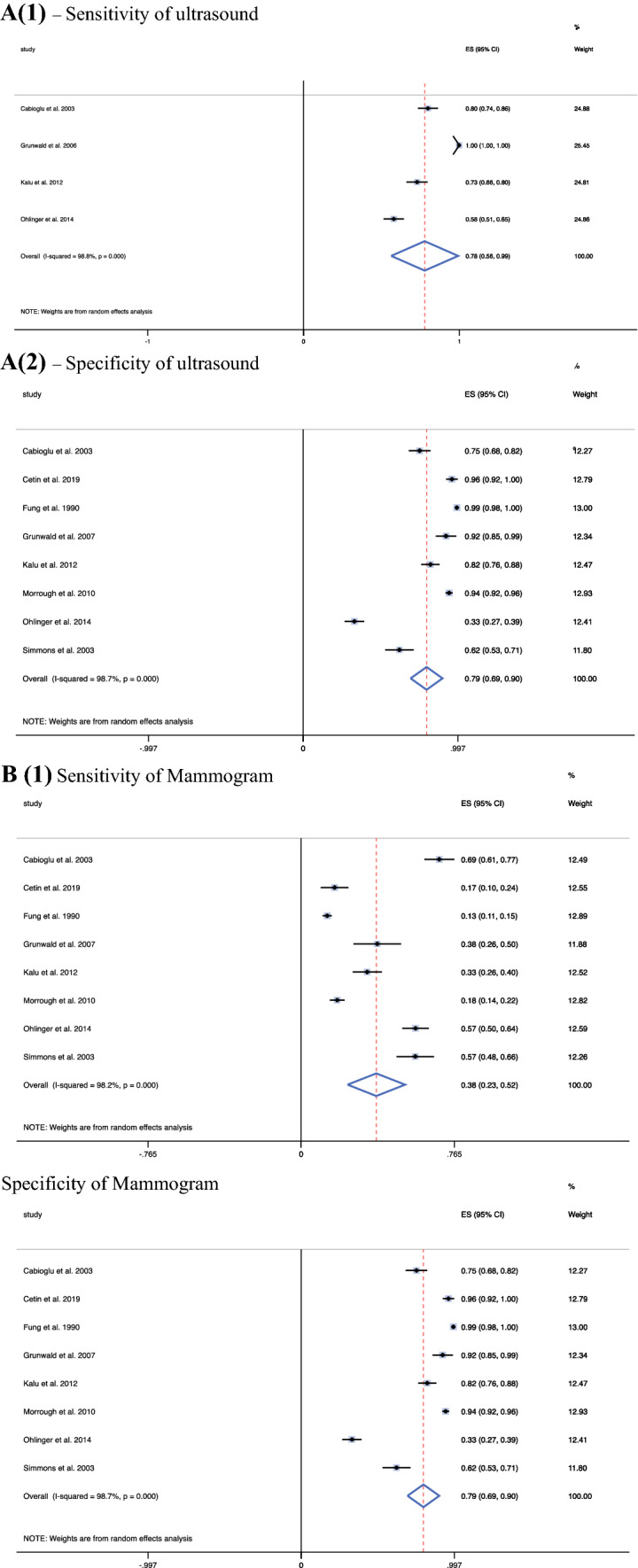

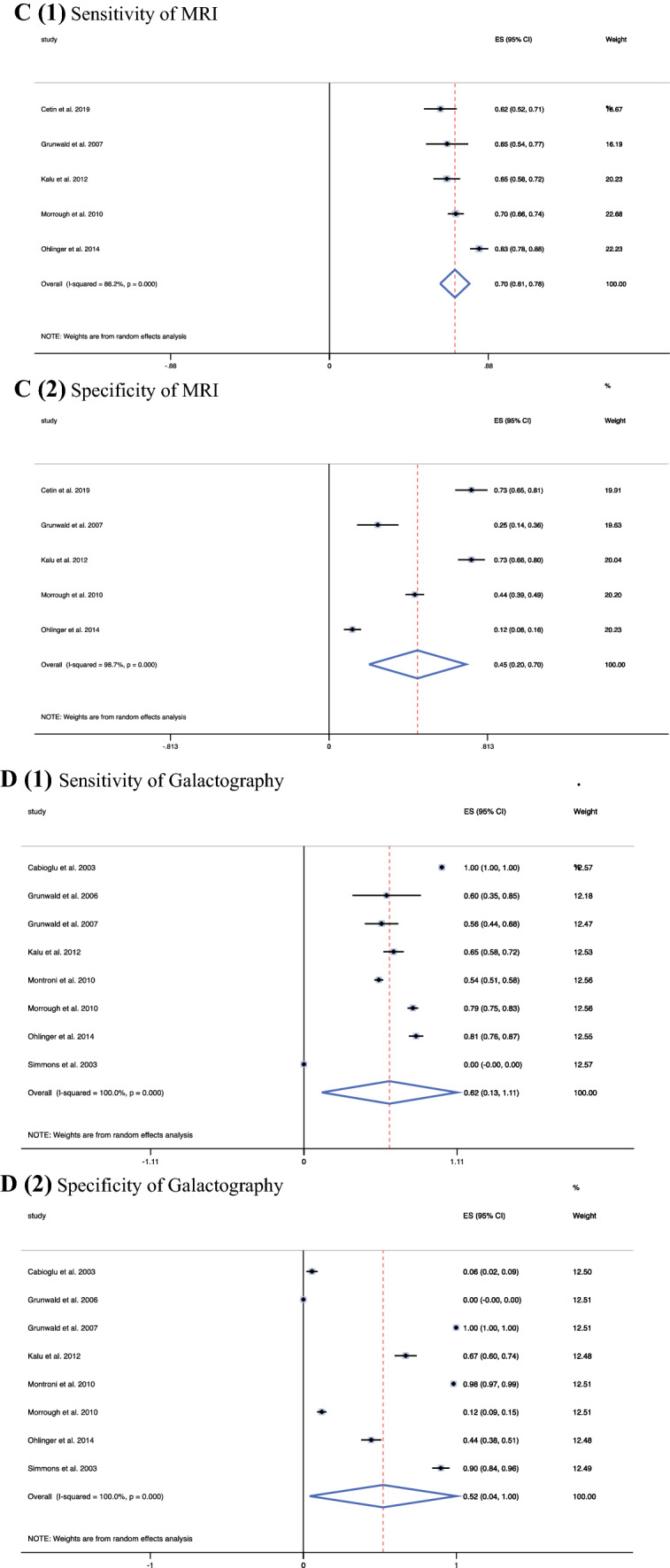
Forest plots depicting the individual pooled sensitivity and specificity of other diagnostic methods: ultrasonography (8 studies), mammography (8 studies), magnetic resonance imaging (MRI) (8 studies), galactrography (8 studies). **A1** Sensitivity of ultrasound. **A2** Specificity of ultrasound. **B1** Sensitivity of mammogram. **B2** Specificity of mammogram. **C1** Sensitivity of magnetic resonance imaging (MRI). **C2** Specificity of MRI. **D1** Sensitivity of galactography. **D2** Specificity of galactography

Other diagnostic methods yielded a range of sensitivities, specificities, and PPVs. Ultrasound was observed to have a pooled sensitivity of 0.70 (95 % CI, 0.60–0.80) and a lower pooled specificity of 0.58 (95 % CI, 0.24–0.75), with a PPV of 0.78 (95 % CI, 0.56–0.99) (Fig. 3A). Mammogram yielded a low pooled sensitivity of 0.38 (95 % CI, 0.23–0.52), a higher pooled specificity of 0.79 (95 % CI, 0.69–0.90), and a PPV of 0.49 (95 % CI, 0.24–0.75) (Fig. 3 B). For MRI, a pooled sensitivity of 0.70 (95 % CI, 0.61–0.78) and a pooled specificity of 0.45 (95 % CI, 0.20–0.70) together with a PPV of 0.57 (95 % CI, 0.55–0.79) were observed (Fig. 3C). Galactography yielded a pooled sensitivity of 0.62 (95 % CI, 0.13–1.11), a pooled specificity of 0.52 (95 % CI, 0.04–1.00), and a PPV of 0.48 (95 % CI, 0.00–0.95) (Fig. 3D). Finally, for blood-stained discharge, the malignancy rate was 0.57 (95 % CI, 0.54–0.60), signifying that 57 % of those presenting with a blood-stained nipple discharge went on to receive a malignant diagnosis. Moreover, the calculated PPV of a blood-stained nipple discharge cytology was 0.27 (95 % CI, 0.17–0.36) (Supplement 1; Fig. 2).

## Discussion

This meta-analysis integrated the diagnostic accuracy of nipple discharge fluid cytology and diagnostic imaging across published clinical studies. The primary finding was that the sensitivity of PND evaluation for the detection of both benign disease and breast cancer is poor. The sensitivity was respectively 75 % (95 % CI, 0.74–0.77) and 62 % (95 % CI, 053–0.71), and the specificity was respectively 87 % (95 % CI, 0.86–0.88) and 71 % (95 % CI, 0.57–0.81). Overall, these specificity and sensitivity data are echoed across individual studies of patients presenting with symptomatic nipple discharge.^16,17^ Interestingly, the diagnostic accuracy of nipple cytologic analysis of patients with PND is similar to that of other diagnostic tests, with sensitivities ranging from a high of 70 % for both ultrasound and MRI to a specificity high of 79 % for mammography. Critically, in the case of a patient whose sole symptom is nipple discharge, no individual diagnostic test, whether imaging or cytologic, yielded a sensitivity or specificity high enough for its use as a stand-alone test.

Interestingly, the presence of blood did not appear to predict a breast cancer diagnosis (PPV, 27 %; 95 % CI, 0.17–0.36), and the high association of blood and malignancy (57 %) may be confounded by studies including only data on patients with blood and malignancy.^18,19^ Therefore, despite reports suggesting the importance of color or presence of blood,^18,20^ the clinical utility of nipple fluid assessment is limited. For both benign and malignant diagnoses, the frequent lack of cellular material makes it difficult to analyze abnormalities. Nipple fluid cytology of the breast is deemed increasingly difficult because cancer cells from the breast tend to be both smaller and less pleomorphic than their counterparts from other parts of the body.^21^ Moreover, cytologic criteria for malignancy are less obvious in nipple discharge smears because they have a tendency to contain degenerated cells.^22^ In addition, interpretation may be subject to inter-reporter variability or relative inexperience, as well as the presence of atypical cellular changes unrelated to a malignancy, leading to either a higher degree of false-positive or false-negative findings.

Despite the challenges associated with nipple cytologic analysis and notwithstanding the small proportion of patients presenting with PND who will go on to receive a breast cancer diagnosis,^23^ it may be the only presenting clinical symptom of a breast cancer and therefore cannot be dismissed. Although cytology is no longer used in some centers, nipple smear cytology continues to be used in clinical practice globally. The rationale behind its use is that the majority of breast cancers arise from the epithelial lining of the terminal ducts and thus are denoted as invasive ductal carcinomas.^24^ Therefore, it is accepted that nipple discharge fluid directly reflects the tumor microenvironment and for high-risk individuals indicates the lead up to cancer.^25^ However, it also has been shown that not all ducts drain to the nipple surface,^26^ suggesting that even if adequate, cytologic analysis could miss a proportion of breast cancers.

A further challenge is the range of cellular findings and whether this is representative of benign or malignant disease. For example, papillary clusters can be a cytologic finding of both benign and malignant pathologies.^27,28^

Because the reviewed diagnostic methods have limited ability to confirm or exclude a breast cancer diagnosis for patients presenting with PND, surgical intervention in the form of a microdochectomy or total duct excision is required for a definitive diagnosis or adequate reassurance. Indeed, the findings of this meta-analysis might suggest that such patients could undergo imaging to exclude mass lesions, including possibly MRI.^29^ However, a large proportion of patients go on to have a microdochectomy because a normal MRI does not exclude an adjacent or underlying malignancy.^29^ Therefore, it may be argued in light of the results from the current meta-analysis that cytology is no longer necessary because it adds very little further diagnostic information. An alternative pathway for the management of single-duct nipple discharge could instead rely on clinical assessment using ultrasound ± mammogram followed by an MRI, with a diagnostic microdochectomy if radiologic findings are unremarkable.

Moreover, our review suggests that no single diagnostic technique can be used in isolation, even amid these changing times, with the need to minimize hospital appointments and unnecessary surgery. It does, however, suggest scope for development of a more comprehensive diagnostic tool to assess nipple discharge. With the explosion of metabolomics during the last decade yielding promising results,^7,30–32^ the interrogation of tiny amounts of fluid such as nipple discharge fluid using newer technology must be investigated, with awareness of the need for high diagnostic accuracy, fast turnaround time, and reproducibility.

The great strength of this meta-analysis was its comprehensive review of nipple cytology diagnostics toward pooled diagnostic accuracy. The decision to include cytology papers from such a large time span was intended to reflect the longevity of the technique’s use and its diagnostic accuracy in the context of evolving diagnostic practices. Moreover, this is the first review to interrogate the use of nipple smear cytology to detect both benign and malignant breast disease and to compare its performance with that of other breast imaging methods. The most recently published comparable review by Filipe et al.^4^ considers only malignant diagnostics and independently compares other imaging methods for which only histopathology is available. In addition, their study overlooked literature from which guidelines were drawn.^33–35^ Comparing other imaging methods and cytology within the same patient cohorts reduces patient selection bias and therefore reflects more accurately on the diagnostic capabilities of each technique in the same settings during the time period.

A potential limitation of the current review was in the quality of the papers retrieved. The QUADAS scoring ranged from 4 to 14 and reflected the variable nature of the study design and its relevance to the review question. For example, the study included papers reporting only the cytology results of patients presenting with bloody nipple discharge who had a cancer diagnosis. It is evident that the sensitivity was falsely elevated because the negative results are not disclosed in the paper.^36^ Similarly, not all papers had a strict definition of what was considered as a pathologic nipple discharge, so higher rates of “physiologic” discharge may have been included within the presenting numbers.

## Conclusions

Pooled data from the included studies demonstrated that the diagnostic accuracy of nipple discharge cytology is limited and has poor sensitivity for symptomatic women. The color of nipple discharge fluid, although yielding a high positive malignancy rate, demonstrated a poor PPV. Emerging technologies for analysis of nipple fluid must have a higher diagnostic accuracy than nipple cytology while offering advantages in terms of cost, reproducibility, user dependency, and turnaround time.

## Supplementary Information

Below is the link to the electronic supplementary material.

Supplementary file1 (DOCX 249 KB)
