# Supplementary material for: Diagnostic Accuracy of Nipple Discharge Fluid Cytology: A Meta-Analysis and Systematic Review of the Literature
Source: Ann Surg Oncol. 2021 Nov 27;29(3):1774–86. doi: 10.1245/s10434-021-11070-2 (PMC8627297; doi:10.1245/s10434-021-11070-2)
Supplement: Supplementary file 1 — Supplementary file1 (DOCX 249 KB) [file 10434_2021_11070_MOESM1_ESM.docx]

Articles identified in systematic search (n=837)

Medline n=278

EMBASE n=459

Scopus n=100

ME

Articles after title/abstract screening (n=530)

Duplicate papers (n=307)

Articles for full-text assessment (n=218)

Articles excluded (173)

- No English translation of article (n=70)
- No diagnostic nipple discharge cytology data (n=45)
- Abstract only (n=15)
- ND not compared to a gold standard (n=14)
- Duplication of dataset (n=12)
- FNA and nipple smear cytology results merged (n=7)
- Nipple discharge not primary presenting symptom (n=2)
- DL fluid cytology but no simple ND cytology (n=2)
- Paper not available (n=2)
- Case report (n=2)
- Male and female cytology mixed (n=1)
- NAF cytology not ND cytology (n=1)

Articles for analysis (n=45)

Articles excluded based on title and abstract (irrelevant or failed inclusion criteria)

(n=312)

Supplement 1, Figure 1: PRISMA

Supplement 1, Table 1: QUADAS-2 Scoring

| Author and Year | Patient Selection | | Index Test(s) | | Reference Standard | | Flow and Timing | Total (max 14) |
| --- | --- | --- | --- | --- | --- | --- | --- | --- |
|  | Risk of bias | Applicability | Risk of bias | Applicability | Risk of bias | Applicability | Risk of bias |  |
| Alcock et al. 2008 | 2 | 2 | 2 | 1 | 2 | 2 | 2 | 13 |
| Bauer et al. 1998 | 2 | 2 | 1 | 1 | 1 | 1 | 0 | 8 |
| Cabioglu et al. 2004 | 2 | 2 | 1 | 1 | 0 | 1 | 1 | 8 |
| Cabioglu et al. 2003 | 2 | 2 | 1 | 1 | 0 | 1 | 1 | 8 |
| Carty et al. 1994 | 2 | 2 | 1 | 1 | 1 | 1 | 1 | 9 |
| Castellano et al. 2017 | 0 | 2 | 1 | 1 | 2 | 1 | 1 | 8 |
| Cetin et al. 2019 | 1 | 2 | 1 | 1 | 1 | 1 | 1 | 8 |
| Cilotti et al. 1996 | 1 | 1 | 2 | 1 | 1 | 1 | 1 | 8 |
| Denewer et al. 2008 | 2 | 2 | 0 | 1 | 0 | 1 | 0 | 6 |
| Dinkel et al. 2001 | 1 | 2 | 2 | 1 | 1 | 2 | 2 | 11 |
| El-Daly et al. 2009 | 1 | 1 | 1 | 2 | 2 | 2 | 2 | 11 |
| Florio et al. 1999 | 2 | 2 | 1 | 1 | 1 | 1 | 1 | 9 |
| Fung et al. 1990 | 2 | 2 | 2 | 1 | 2 | 1 | 2 | 12 |
| Funovics et al. 2002 | 2 | 1 | 1 | 1 | 1 | 1 | 1 | 8 |
| Groves et al. 1996 | 1 | 1 | 1 | 1 | 1 | 1 | 1 | 7 |
| Grunwald et al. 2006 | 1 | 1 | 1 | 1 | 1 | 1 | 1 | 7 |
| Grunwald et al. 2007 | 0 | 1 | 1 | 1 | 0 | 0 | 1 | 4 |
| Haan et al. 2009 | 2 | 2 | 2 | 2 | 1 | 1 | 1 | 11 |
| Hou et al. 2000 | 2 | 2 | 2 | 2 | 2 | 2 | 2 | 14 |
| Hou et al. 2002 | 2 | 2 | 2 | 2 | 2 | 2 | 1 | 13 |
| Hunerbein et al. 2007 | 1 | 1 | 1 | 1 | 1 | 1 | 1 | 7 |
| Kalu et al. 2012 | 1 | 2 | 2 | 2 | 2 | 2 | 2 | 13 |
| Kan et al. 2018 | 1 | 1 | 1 | 1 | 1 | 1 | 1 | 7 |
| Kaplan et al. 2011 | 2 | 2 | 2 | 2 | 1 | 1 | 2 | 12 |
| Kjellgren et al. 1956 | 1 | 1 | 1 | 2 | 1 | 2 | 1 | 9 |
| Kooistra et al. 2009 | 2 | 2 | 2 | 2 | 2 | 2 | 1 | 13 |
| Kuroi et al. 1997 | 1 | 1 | 1 | 1 | 1 | 1 | 2 | 8 |
| Lanitis et al. 2008 | 1 | 1 | 1 | 1 | 2 | 1 | 2 | 9 |
| Lee et al. 2002 | 1 | 0 | 2 | 1 | 1 | 1 | 0 | 6 |
| Leis et al. 1973 | 2 | 2 | 1 | 2 | 2 | 2 | 2 | 13 |
| Markopoulos et al. 2006 | 1 | 1 | 1 | 1 | 0 | 1 | 1 | 6 |
| Matsunaga et al. 2008 | 2 | 1 | 2 | 2 | 1 | 1 | 1 | 10 |
| Montroni et al. 2010 | 2 | 1 | 2 | 1 | 2 | 2 | 2 | 12 |
| Morrough et al. 2010 | 1 | 1 | 0 | 1 | 2 | 2 | 1 | 8 |
| Ohlinger et al. 2014 | 2 | 1 | 2 | 1 | 1 | 1 | 2 | 10 |
| Pritt et al. 2004 | 2 | 1 | 2 | 2 | 1 | 1 | 1 | 10 |
| Rimsten et al. 1976 | 0 | 1 | 1 | 1 | 1 | 1 | 1 | 6 |
| Simmons et al. 2003 | 1 | 1 | 1 | 1 | 1 | 1 | 1 | 7 |
| Shen et al. 2001 | 0 | 1 | 0 | 1 | 0 | 1 | 1 | 4 |
| Walker et al. 1958 | 0 | 1 | 2 | 1 | 1 | 1 | 1 | 7 |
| Yang et al. 2014 | 1 | 1 | 0 | 0 | 1 | 1 | 1 | 5 |
| Jacobs et al. 2005 | 1 | 0 | 1 | 1 | 1 | 1 | 1 | 6 |
| Yammamoto et al. 2001 | 1 | 1 | 1 | 2 | 1 | 0 | 1 | 7 |
| Zervoudis et al. 2014 | 2 | 1 | 1 | 0 | 1 | 2 | 1 | 8 |
| Ciatto et al. 1986 | 2 | 1 | 1 | 1 | 0 | 1 | 0 | 6 |

Supplement 1, Figure 2: PPV of blood-stained discharge

Supplement 2, Table 1: Malignant Cytology (Cn4/5)

| Author and year | No of patients | No of samples | Malignant spec | Malignant sens | Malignant PPV |
| --- | --- | --- | --- | --- | --- |
| Alcock et al. 2010 (37) | 49 | 49 | 0.127 | 0 | 0.6 |
| Bauer et al. 1998 (38) | 12 | 23 | 0.02 | 0.17 |  |
| Cabioglu et al. 2004 (39) | 188 | 23 |  | 0.087 | 1 |
| Cabioglu et al. 2003 (20) | 146 | 69 | 0.81 | 0.27 | 0.29 |
| Carty et al. 1994 (40) | 56 | 56 |  | 0 |  |
| Castellano et al. 2017 (68) | 139 | 139 | 1 | 0.51 | 1 |
| Cetin et al. 2019 (41) | 111 | 95 | 36.3 | 76.9 |  |
| Ciatto et al. 1986 (42) | 50181 | 3687 | 0.89 | 0.61 | 0.63 |
| Cilotti et al. 1996 (43) | 67 | 67 | 0.45 | 1 | 0.22 |
| Denewer et al. 2008 (44) | 54 | 54 | 0.8 | 0.4 | 0.33 |
| Dinkel et al. 2001 (16) | 384 | 384 | 0.97 | 0.31 | 0.55 |
| El-Daly et al. 2009 (18) | 98 | 98 | 1 | 0.5 | 1 |
| Florio et al. 1999 (45) | 1251 | 194 | 0 | 100 | 0.28 |
| Fung et al. 1990 (46) | 840 | 176 | 0.7 | 0.83 | 0.63 |
| Funovics et al. 2003 (47) | 134 | 134 | 0.72 | 0.26 | 0.54 |
| Groves et al. 1996 (48) | 338 | 329 | 99.5 | 46.5 | 87.5 |
| Grunwald et al. 2006 (49) | 15 | 15 | 0 | 0.33 | 1 |
| Grunwald et al. 2007 (67) | 64 | 58 | N/A | N/A | N/A |
| Hahn et al. 2009 (50) | 33 | 32 | N/A | N/A | N/A |
| Hou et al. 2000 (51) | 146 | 156 | 0.89 | 0.53 | 0.47 |
| Hou et al. 2002 (52) | 487 | 176 | 0.9 | 0.52 | 0.48 |
| Hunerbein et al. 2007 (53) | 101 | 45 | 1 | 1 | 100 |
| Jacobs et al. 2005 (54) | 11 | 8 | 0.67 | 0.50 | 0.50 |
| Kalu et al. 2012 (55) | 160 | 89 | 0.30 | 0.75 | 0.68 |
| Kan et al. 2018 (17) | 102 | 37 |  |  |  |
| Kaplan et al. 2011 (19) | 50 | 50 | 0.94 | 0.10 | 0.33 |
| Kjellgren et al. 1956 (56) | 39 | 39 | 0.61 | 0.67 | 0.17 |
| Kooistra et al. 2009 (12) | 618 | 618 | 0.96 | 0.10 | 0.25 |
| Kuroi et al. 1997 (57) | 19 | 19 | 0.00 | 100 | 83.3 |
| Lanitis et al. 2008 (29) | 76 | 76 | 0.00 | 0.00 | 0.00 |
| Lee et al. 2002 (58) | 165 | 174 | 1.00 | 0.56 | 1.00 |
| Leis et al. 1973 (35) | 259 | 259 | 0.96 | 0.75 | 0.73 |
| Markopoulos et al. 2006 (36) | 110 | 110 |  | 100 | 40 |
